# Norovirus Recombinant Strains Isolated from Gastroenteritis Outbreaks in Southern Brazil, 2004–2011

**DOI:** 10.1371/journal.pone.0145391

**Published:** 2016-04-26

**Authors:** Tulio Machado Fumian, Juliana da Silva Ribeiro de Andrade, José Paulo Gagliardi Leite, Marize Pereira Miagostovich

**Affiliations:** Laboratory of Comparative and Environmental Virology, Oswaldo Cruz Institute, Oswaldo Cruz Foundation, Rio de Janeiro, Brazil; University of Malaya, MALAYSIA

## Abstract

Noroviruses are recognized as one of the leading causes of viral acute gastroenteritis, responsible for almost 50% of acute gastroenteritis outbreaks worldwide. The positive single-strand RNA genome of noroviruses presents a high mutation rate and these viruses are constantly evolving by nucleotide mutation and genome recombination. Norovirus recombinant strains have been detected as causing acute gastroenteritis outbreaks in several countries. However, in Brazil, only one report of a norovirus recombinant strain (GII.P7/GII.20) has been described in the northern region so far. For this study, 38 norovirus strains representative of outbreaks, 11 GII.4 and 27 non-GII.4, were randomly selected and amplified at the ORF1/ORF2 junction. Genetic recombination was identified by constructing phylogenetic trees of the polymerase and capsid genes, and further SimPlot and Bootscan analysis of the ORF1/ORF2 overlap. Sequence analysis revealed that 23 out of 27 (85%) non-GII.4 noroviruses were recombinant strains, characterized as: GII.P7/GII.6 (n = 9); GIIP.g/GII.12 (n = 4); GII.P16/GII.3 (n = 4); GII.Pe/GII.17 (n = 2); GII.P7/GII.14 (n = 1); GII.P13/GII.17 (n = 1); GII.P21/GII.3 (n = 1); and GII.P21/GII.13 (n = 1). On the other hand, among the GII.4 variants analyzed (Den Haag_2006b and New Orleans_2009) no recombination was observed. These data revealed the great diversity of norovirus recombinant strains associated with outbreaks, and describe for the first time these recombinant types circulating in Brazil. Our results obtained in southern Brazil corroborate the previous report for the northern region, demonstrating that norovirus recombinant strains are circulating more frequently than we expected. In addition, these results emphasize the relevance of including ORF1/ORF2-based analysis in surveillance studies as well as the importance of characterizing strains from other Brazilian regions to obtain epidemiological data for norovirus recombinant strains circulating in the country.

## Introduction

Noroviruses (NoV) are members of the *Caliciviridae* family, and is now recognized as one of the leading causes of acute gastroenteritis (AGE), responsible for almost 50% of AGE outbreaks worldwide [1,2]. NoV are primarily associated with outbreaks of AGE in semi-closed settings such as elderly care facilities, hospitals, cruise ships and childcare centers [2,3]. These epidemics have occurred globally since the mid-1990s with increasing frequency [4,5]. Consequently, NoV-associated AGE has become a major public health concern for which there is no available anti-viral agent or preventative vaccine yet available.

NoV present a positive-polarity RNA genome of approximately 7500 nucleotides (nt) in length, presenting a high mutation rate and high genetic variability; it is organized as three open reading frames (ORFs), with ORF1 and ORF2 overlapping by about 20 nt [6,7]. ORF1 encodes non-structural proteins including RNA-dependent RNA polymerase (RdRp). ORF2 encodes a major capsid protein (VP1) that contains an N-terminal arm, a shell or S-domain and a protrusion or P-domain, and ORF3 encodes a minor capsid protein (VP2); both proteins are translated from subgenomic RNA [8]. NoV have been classified into six genogroups (GI to GVI) based on VP1 amino acid sequence [9]. Each genogroup can be further divided into genotypes, and at least 36 genotypes are recognized to date [10–12]. NoV are in constant evolution, with new strains frequently arising due to nucleotide point mutation (antigenic drift) and genetic recombination during a co-infection [13]. Recombination is one of the main driving forces shaping the evolution of viruses, providing a mechanism for generating antigenically novel viruses and, therefore, the ability to evade the immune system [13,14]. In the NoV genome, a recombination hotspot is present near the ORF1/ORF2 junction and a variety of recombinant strains have been detected worldwide [7,13,15–18].

In Brazil, the role of NoV as causative agents of AGE causing outbreaks, sporadic cases, and hospitalization are well documented [19–23]. However, there is a lack of data concerning knowledge of the circulation of NoV recombinant strains in the Brazilian population, since only one report demonstrated a recombinant strain (GII.P7/GII.20) in a community of African descent in northern Brazil [24].

Recently, it was demonstrated the importance of NoV in AGE outbreaks in Southern Brazil, but genotype characterization was performed based only on capsid gene sequences [19]. In the present study, we aimed to investigate the occurrence of recombination in NoV strains associated with AGE outbreaks in the Rio Grande do Sul state (southern region of Brazil) between 2004 and 2011. The recombinant strains were identified by sequence analysis of the ORF1/ORF2 junction region, followed by SimPlot and Bootscan analysis.

## Materials and Methods

### Ethics statement

AGE surveillance is performed through a hierarchical network in which samples are provided by medical request in hospitals and health centers, monitored by the Brazilian Unified Health System (SUS). Fecal samples were collected by the state Central Laboratory and then forwarded to the Laboratory of Comparative and Environmental Virology, Oswaldo Cruz Institute (FIOCRUZ), Ministry of Health. Forms with epidemiological and clinical data accompanied each fecal sample. No patient information was used other than to determine city residence or possible association with outbreaks, and data were maintained anonymously and securely. This study is part of a project that covers diagnosis, surveillance and molecular epidemiology of viruses that cause AGE, approved by the Ethics Committee of FIOCRUZ (CEP No. 311/06).

### Clinical samples

NoV-positive stool samples were collected and analyzed during a retrospective study, as reported previously, that aimed to describe the role of these viruses in causing AGE outbreaks which occurred in the state of Rio Grande do Sul, southern Brazil, in a period of eight years (2004–2011) [19]. For this study, 38 NoV strains representative of outbreaks, 11 GII.4 and 27 non-GII.4, were selected randomly and amplified at the ORF1/ORF2 junction (524nt).

### RNA extraction and cDNA synthesis

Viral RNA was purified from stool samples stored at –20°C. A 140 μL suspension (10% w/v) of each stool sample was prepared with Tris-calcium buffer (pH = 7.2) and subjected to an automatic RNA extraction procedure using a QIAamp^®^ Viral RNA Mini kit (QIAGEN, CA, USA) and a QIAcube^®^ automated system (QIAGEN), according to the manufacturer’s instructions. Part of the isolated nucleic acid was transcribed to cDNA using a High Capacity cDNA Reverse Transcription Kit (Life Technologies™, NY, USA), and an aliquot was immediately stored at −80°C. In each extraction procedure, RNAse/DNAse-free water was used as negative control.

### Norovirus genotyping

PCR was performed using primers targeting the ORF1/2 junction region, Mon 431/432 [25] and G2SKR [26], to generate 544 bp amplicons. The reaction was performed in a 50 μL mixture of 10 μL cDNA, 5 U Platinum^®^ *Taq* DNA Polymerase (Life Technologies™), and 250 nM of each primer. PCR amplification was performed with an initial denaturation at 95°C for 5 min, followed by 40 cycles of denaturation at 95°C for 30 s, annealing at 50°C for 30 s, extension at 72°C for 1 min, and a final extension at 72°C for 10 min. The amplicons obtained were purified using a QIAquick PCR Purification Kit (QIAGEN, Valencia, CA, USA) following the manufacturer’s recommendations. For DNA sequencing, the purified products were sent to the FIOCRUZ Institutional Platform for DNA sequencing (PDTIS), performed using an ABI Prism BigDye Terminator Cycle Sequencing Ready Reaction Kit^®^ and ABI Prism 3730 Genetic Analyzer (both from Applied Biosystems, Foster City, CA, USA). Following chromatogram analysis, consensual sequences were obtained using BioEdit [27]. Initially, NoV genotypes were assigned using an online genotyping tool (http://www.rivm.nl/mpf/norovirus/typingtool) [11] and the strains were named, with the genotype of the polymerase indicated with an uppercase letter p, as proposed by Kroneman et al. [12].

### Recombination analysis

After results were obtained from the genotyping tool, two sequence datasets were constructed, one considering the region coding RdRp (partial ORF1) and the other including the capsid coding region (partial ORF2). Comparable sequences containing ORF1/ORF2 overlap for different NoV genotypes were retrieved from the National Center for Biotechnology Information (NCBI) database. Phylogenetic analysis was performed for both datasets including comparable sequences of different NoV genotypes. Phylogenetic trees were constructed using the neighbor-joining method (Kimura two-parameter model, 2000 bootstrap replications for branch support) in MEGA 6.0 [28]. To further confirm putative recombinant strains and to identify a putative recombination point according with previous reports [13], plot similarity was carried out using SimPlot version 3.5.1 [29]. SimPlot analysis was performed by setting the window width and the step size to 200 bp and 20 bp, respectively. Different methods implemented in the Recombination Detection Program v.4.16 (RDP4) were also used [30], such as Bootscan/Recscan analysis. The sequences obtained in the present study were included as queries, while putative parental sequences were obtained from the GenBank database, and recombinant strains were confirmed with significant events (*p* < 0.01).

### GenBank accession numbers

The nucleotide sequences obtained in this study were submitted to the NCBI (GenBank, http://www.ncbi.nlm.nih.gov/) and received accession numbers KR074148–KR074191.

## Results

**Fig 1:**
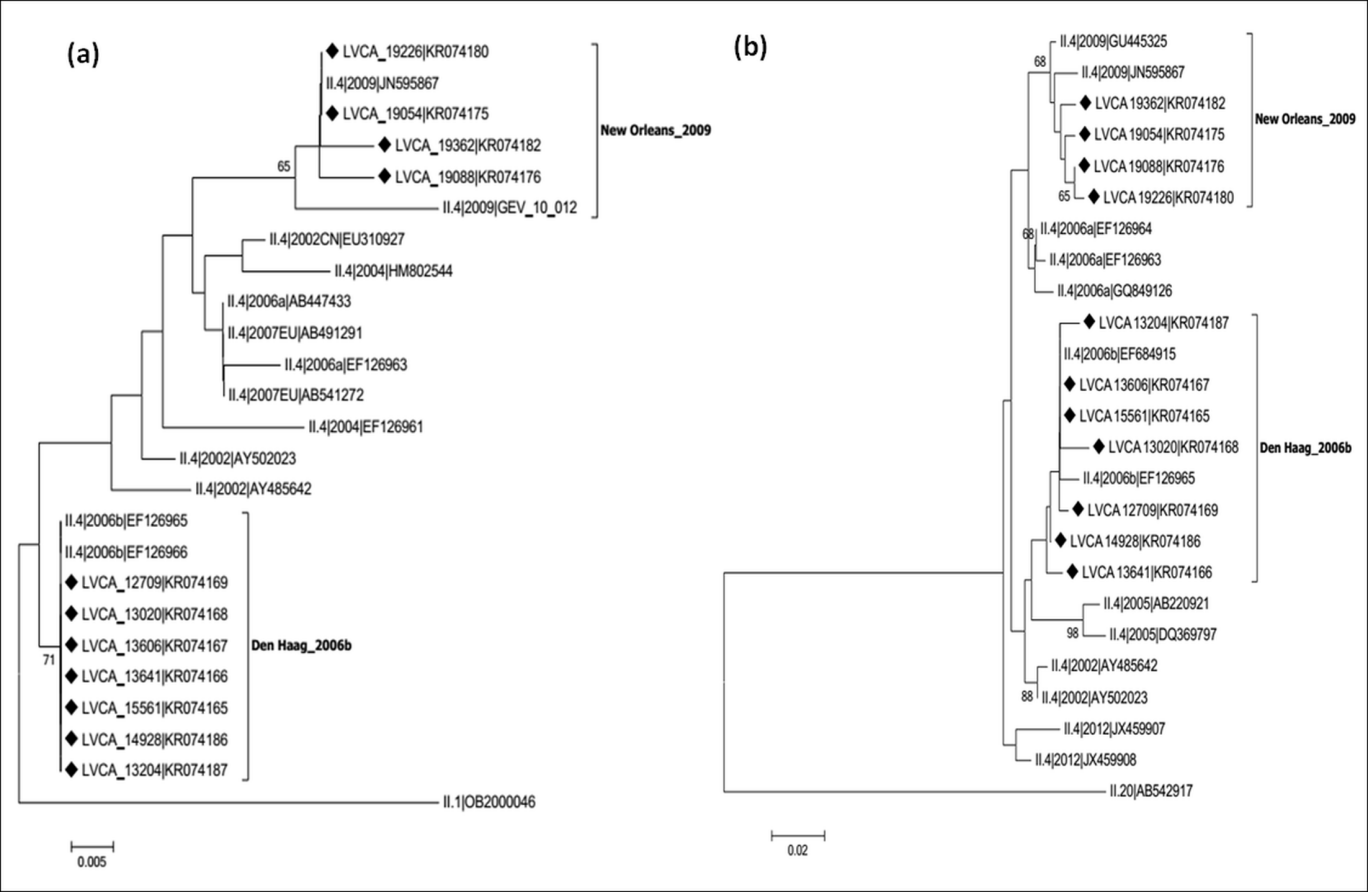
Phylogenetic analyses of NoV GII.4 sequences based on the polymerase region (ORF1) and capsid region (ORF2). (a) Phylogenetic tree of 231 bp within the polymerase region (3’ ORF1). (b) Phylogenetic tree of 277 bp within the capsid region (5’ ORF2). References strains of NoV GII.4 variants are named according to GenBank with their respectively accession numbers. Brazilian GII.4 strains are marked with a filled diamond. The scale bar at the bottom of the tree indicates distance. Bootstrap values (2,000 replicates) are shown at the branch nodes and values lower than 60% are not shown.

**Fig 2:**
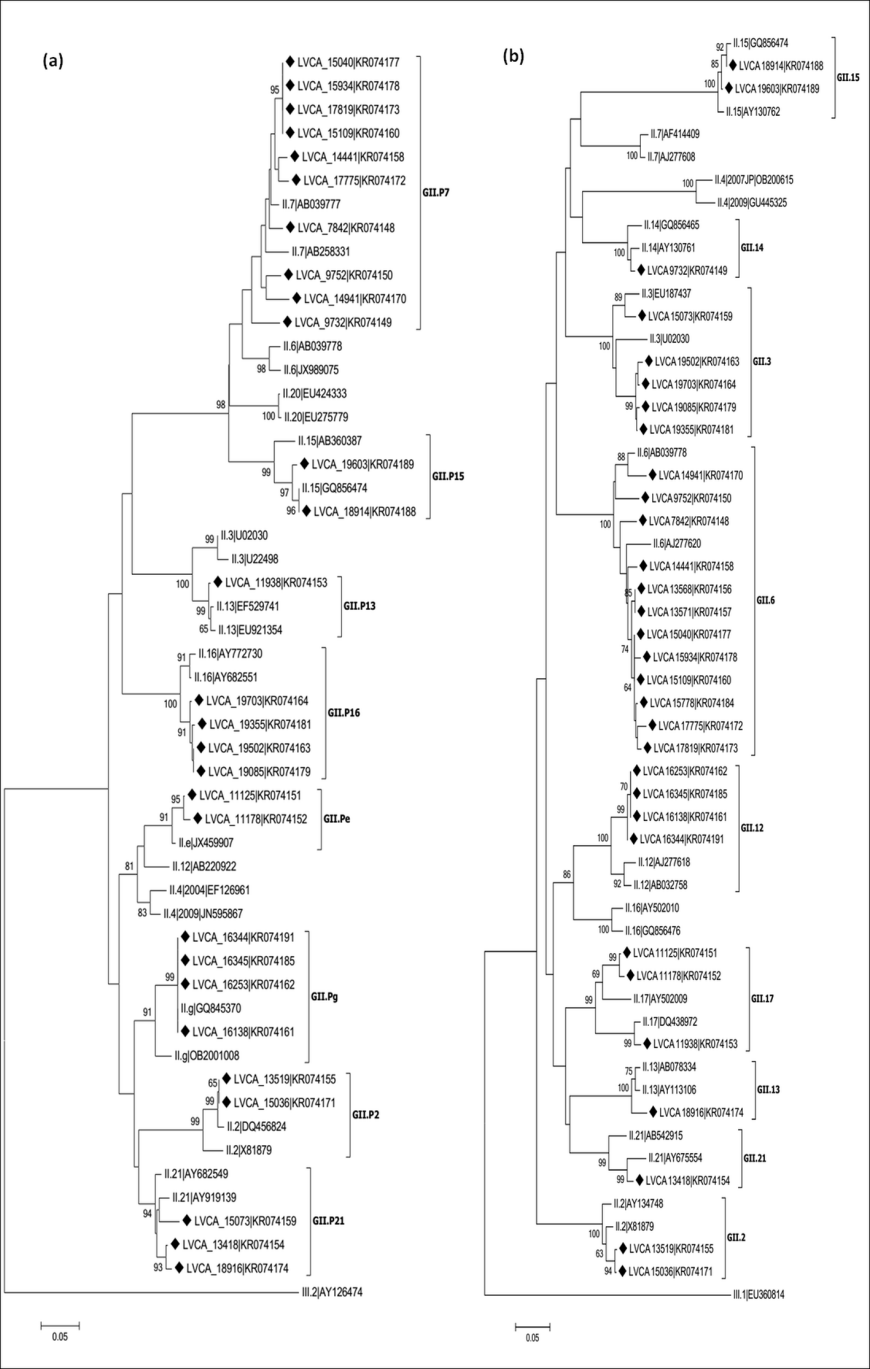
Phylogenetic analyses of NoV GII sequences based on the polymerase and capsid regions. (a) Phylogenetic tree of 231 bp within the polymerase region (3’-ORF1). (b) Phylogenetic tree of 277 bp within the capsid region (5’-ORF2). References strains of NoV genotypes are named according to GenBank with their respectively accession numbers. Brazilian GII.4 strains are marked with a filled diamond. The scale bar at the bottom of the tree indicates distance. Bootstrap values (2000 replicates) are shown at the branch nodes and values lower than 60% are not shown.

NoV recombinant genotypes were characterized in 23 out of 27 (85%) of the non-GII.4 samples, identified by constructing phylogenetic trees of polymerase and capsid genes, and further SimPlot analysis of the ORF1/ORF2 overlap (Figs 1 and 2). NoV recombinant strains were identified as: GII.P7/GII.6 (n = 9); GIIP.g/GII.12 (n = 4); GII.P16/GII.3 (n = 4); GII.Pe/GII.17 (n = 2); GII.P7/GII.14 (n = 1); GII.P13/GII.17 (n = 1); GII.P21/GII.3 (n = 1); and GII.P21/GII.13 (n = 1) (Table 1).

**Table 1:** NoV recombinant sequences detected in Southern Brazil during the period of 2004–2011, with the partial 3’-ORF1 and 5’-ORF2 of NoV genome.

Year	Sample identification	Accession number	Acute gastroenteritis outbreak date(month/year)	NoV Genotyping		Bootscan *p*-value
				ORF1	ORF2	
2004	LVCA-7842	KR074148	12/2004	GII.P7	GII.6	6.027 x 10^−4^
	LVCA-9732	KR074149	09/2004	GII.P7	GII.14	3.610 x 10^−4^
	LVCA-9752	KR074150	09/2004	GII.P7	GII.6	9.343 x 10^−3^
2005	LVCA-11125	KR074151	08/2005	GII.Pe	GII.17	4.141 x 10^−5^
	LVCA-11178	KR074152	09/2005	GII.Pe	GII.17	6.426 x 10^−5^
2006	LVCA-11938	KR074153	03/2006	GII.P13	GII.17	8.085 x 10^−9^
2007	LVCA-14441	KR074158	10/2007	GII.P7	GII.6	7.127 x 10^−4^
2008	LVCA-14941	KR074170	04/2008	GII.P7	GII.6	8.509 x 10^−4^
	LVCA-15040	KR074177	04/2008	GII.P7	GII.6	2.093 x 10^−5^
	LVCA-15073	KR074159	04/2008	GII.P21	GII.3	8.195 x 10^−7^
	LVCA-15109	KR074160	05/2008	GII.P7	GII.6	2.807 x 10^−4^
	LVCA-15934	KR074178	11/2008	GII.P7	GII.6	5.513 x 10^−5^
2009	LVCA-16138	KR074161	01/2009	GII.Pg	GII.12	1.680 x 10^−4^
	LVCA-16253	KR074162	03/2009	GII.Pg	GII.12	1.255 x 10^−4^
	LVCA-16344	KR074191	04/2009	GII.Pg	GII.12	8.017 x 10^−5^
	LVCA-16345	KR074185	04/2009	GII.Pg	GII.12	3.012 x 10^−4^
2010	LVCA-17775	KR074172	03/2010	GII.P7	GII.6	5.943 x 10^−4^
	LVCA-17819	KR074173	03/2010	GII.P7	GII.6	1.242 x 10^−4^
	LVCA-18916	KR074174	09/2010	GII.P21	GII.13	7.077 x 10^−11^
	LVCA-19085	KR074179	11/2010	GII.P16	GII.3	8.609 x 10^−11^
2011	LVCA-19355	KR074181	01/2011	GII.P16	GII.3	4.847 x 10^−10^
	LVCA-19502	KR074163	01/2011	GII.P16	GII.3	7.580 x 10^−11^
	LVCA-19703	KR074164	03/2011	GII.P16	GII.3	8.050 x 10^−10^

**Fig 3:**
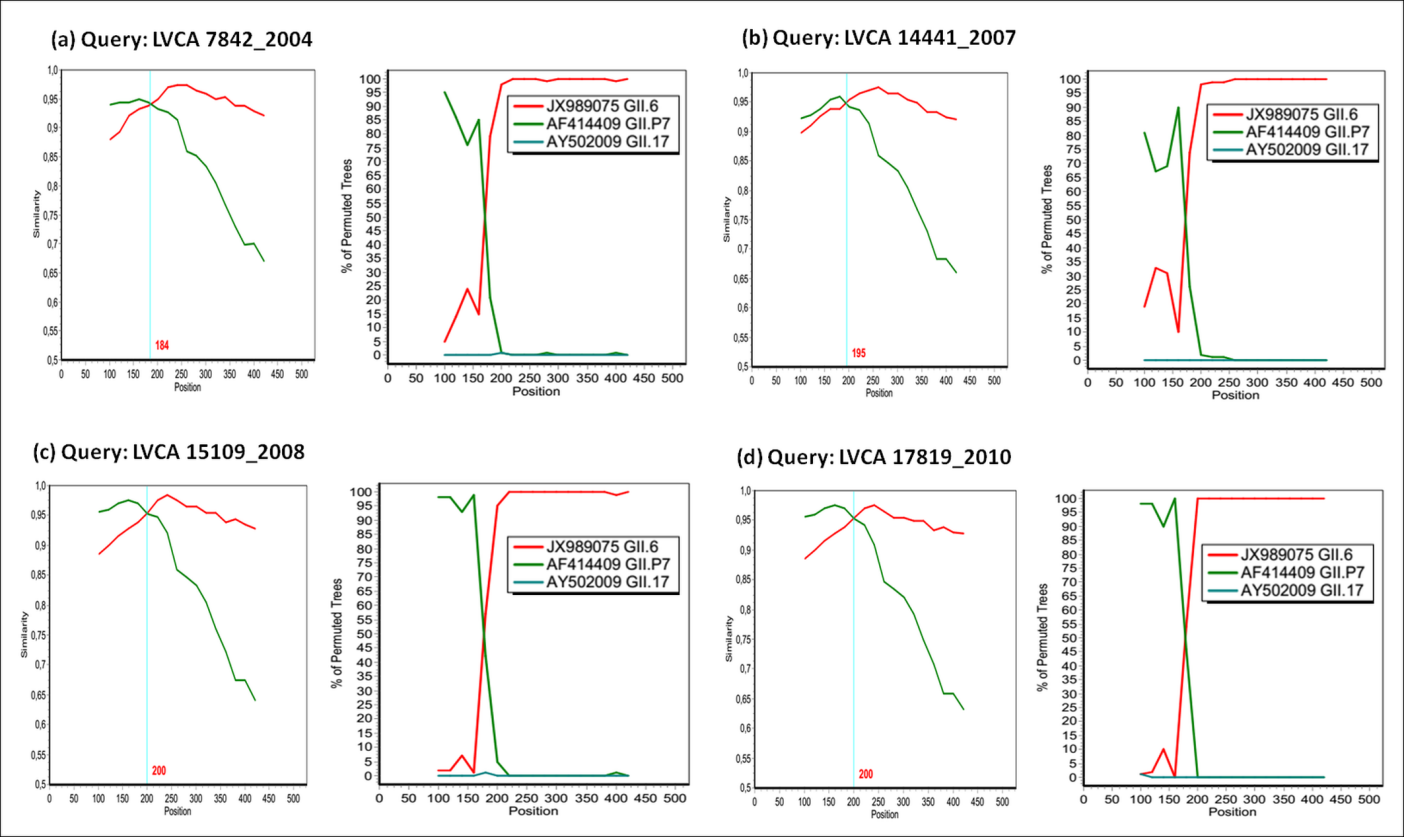
Simplot and Bootscan analyses of the NoV recombinant GII.P7/GII.6 detected in four different years. (a) LVCA7842, (b) LVCA14441, (c) LVCA15109, and (d) LVCA17819. For similarity plot, the y-axis gives the percentage of identity within a sliding window of 200 bp wide, with a step size between plots of 20 bp. The site where the two NoV parental strains of genotypes GII.6 (JX989075) and GII.7 (AF414409) have equal identity to the recombinant (crossed by the vertical blue lines) is the predicted site of recombination. For Bootscan, the y-axis gives the percentage of bootstrap support values of permutated trees using a sliding window of 200 bp wide with a step size between plots of 20 bp. GII.17 strain (AY502009) was used as an outlier sequence.

**Fig 4:**
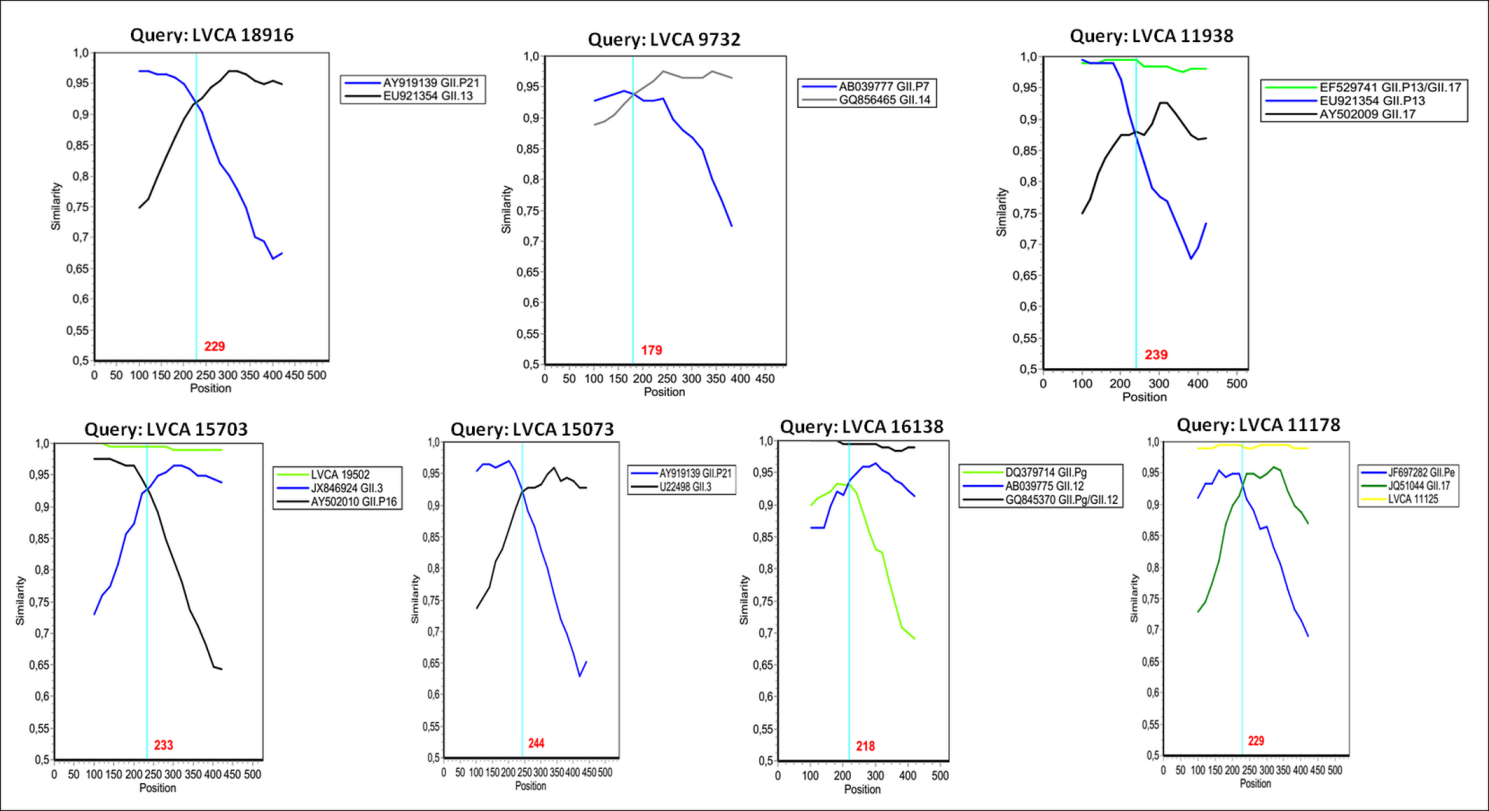
Similarity plots of the NoV recombinant types detected in Southern Brazil during the 2004–2011 period. SimPlot was constructed using Simplot version 3.5.1 with a slide window width of 200 bp and a step size of 20 bp. The vertical axis indicates nucleotide identities (%) between the query sequences (Brazilian samples) and the reference strains. NoV parental strains are indicated by the accession numbers and their respectively ORF1-based genotypes (indicated by the letter P) or ORF2-based genotypes.

SimPlot and Bootscan analysis showed recombination breakpoints near the ORF1/2 junction for all samples (Figs 3 and 4). The most detected recombinant type was GII.P7/GII.6, with strains detected in four years (2004, 2007, 2008, and 2010). Among these nine samples, nucleotide identity ranged from 92% to 99%. We performed SimPlot and Bootscan analysis of four GII.P7/GII.6 strains (one sample for each year) and the recombination breakpoints were detected at positions varying from nucleotides 184 to 200, corresponding to nucleotides 5022–5038 in relation to the reference strain Lordsdale (accession number X86557), localized in the ORF1/2 junction. The location of the recombination breakpoint, near the ORF junction, was similar in all recombinant samples (Figs 3 and 4).

The recombinant types GII.Pe/GII.17 and GII.Pg/GII.12 were detected only in 2005 and 2009, respectively. The four detected samples characterized as GII.Pg/GII.12 showed a high nucleotide identity (> 99%), and were detected in the months of January, March and April. Both ORF1 and ORF2 genotypes of the two samples GII.Pe/GII.17 clustered together in the phylogenetic tree with a nucleotide identity of 99% in the region analyzed. Interestingly, the recombinant GII.P13/GII.17, detected in the following year, has an ORF2 genotype grouped in a separate cluster compared with the sample GII.Pe/GII.17. Samples belonging to the recombinant GII.P16/GII.3 were detected in 2010 and 2011, with genotypes characterized from both regions grouping in the same cluster. The sample LVCA_15073 (GII.P21/GII.3) presented distinct genotypes (both ORF1 and ORF2) that grouped in a separate cluster, when compared with samples GII.P21/GII.13 and GII.P16/GII.3. The four NoV non-recombinant samples were genotyped as GII.P2/GII.2 (n = 2), and GII.P15/GII.15 (n = 2) (Fig 2).

Concerning the 11 NoV GII.4 strains analyzed, no recombination type was identified. GII.4 variants detected were Den Haag_2006b and New Orleans_2009, in both regions analyzed (3’-ORF1 and 5’-ORF2) (Fig 2). These GII.4 samples were detected from 2006 to 2011, and strains shared nucleotide sequence identities between 95% and 99%. Samples belonging to the Den Haag_2006b variant were collected for three years (2006–2008), and samples belonging to the New Orleans_2009 variant were detected in 2010 and 2011. Among samples of both variant groups, nucleotide sequence identities ranged from 98% to 99%.

## Discussion

For the first time in Brazil, we are reporting eight different NoV recombinant strains responsible for AGE outbreaks in the southern region from 2004 to 2011. We observed a high prevalence of NoV recombinant strains (85%) among the non-GII.4 samples analyzed.

Genetic recombination is a widespread phenomenon in NoV, which has a major impact on their evolution and genotype diversity, and has been associated with the emergence of new genotypes. As most NoV recombination occurs in a single hotspot breakpoint located in the ORF1/ORF2 overlap [13,31], a combined characterization of both the polymerase and capsid regions is important to monitor new NoV genotype emergence and recombinant strains [32–35]. Our results emphasize the importance of including the characterization of both regions in surveillance studies since it is probable that the real magnitude of NoV recombination is underestimated in other Brazilian regions and equally in other countries.

The GII.P7/GII.6 was the most frequent recombinant and, unlike the other detected recombinant strains, had a long period of circulation (2004–2010). The GII.P7 genotype was associated with the GII.14 capsid genotype in 2004. In a study performed in a semi-closed community of African descent in northern Brazil in 2008, GII.P7 was described as a recombinant GII.P7/GII.20 detected in a stool sample of a child with NoV-associated gastroenteritis [24]. Also in Brazil, the NoV genotype GII.6 was detected in southeastern and northeastern regions in 2003–2005 and 2007–2008 [36–38]. This recombinant type (GII.P7/GII.6) was first described in 2011 in Burkina Faso [39]. GII.P7/GII.6 recombinants were also reported in other countries such as Italy, Finland, China, and South Africa [34,40–44]. In South America, this recombinant was reported between 2011 and 2012, in diarrheic stool and vomit samples from Uruguayan patients [35]. Due to the high nucleotide similarity between the Uruguayan and Brazilian samples (93%–97%), and taking into account that the Brazilian state of Rio Grande do Sul borders Uruguay, it is possible that the same recombinant strain has circulated in both countries.

The recombinant GII.Pg/GII.12 was the second most frequent found in the present study, although it was detected only in 2009. In that same year in Brazil, the genotype GII.12 was observed circulating in two different regions: northeastern and southeastern [37]; however, as the polymerase genotype was not characterized, we cannot affirm that these samples share the same genetic recombination type. The recombinant GII.Pg/GII.12 was described for the first time in Australia in 2008, from sporadic AGE cases, and in the same year causing outbreaks in New Zealand [33]. In the period 2009–2010, the recombinant GII.Pg/GII.12 had spread worldwide, and was mainly associated with AGE outbreaks [18,31,41,45–49]. As stated by Sang et al., the global spread of GII.Pg/GII.12 strains could be associated with their high evolution rates compared to rates observed for GII.4 [32]. In 2012, this recombinant was reported for the first time in the African continent [34].

Here, we also detected the capsid genotype GII.17 associated with two distinct genotypes, GII.Pe (n = 2) and GIIP.13 (n = 1) in 2005 and 2006, respectively. As they were grouped in a separate phylogenetic cluster, this could indicate that two lineages of the GII.17 genotype may be circulating in Brazil. The genotype GII.17 was also detected in northern Brazil in 2005 and 2009 in the states of Acre and Pará, respectively [22,37]. Globally, GII.Pe has been described as a recombinant in association with other capsid genotypes such as: GII.2, GII.3, GII.4 and GII.12 [33,40,47]. In 2012, the emergence of the new pandemic NoV GII.4 variant Sydney_2012 as a recombinant form (GII.Pe/GII.4) was reported, which led to an increase in NoV activity and associated AGE epidemics in countries including Australia, New Zealand, France, Japan, China and the United States [17,50–52]. The emergence of the pandemic GII.4 variant, which originated from genome recombination, highlights the significance of an antigenic shift on NoV evolution. The capsid genotype GII.17 is described as a recombinant strain in association with the most common GII.P13 ORF1 genotype, although other recombinant forms have been identified such as GII.P16, GII.P3 and GII.P4 [53]. The two samples characterized as recombinant GII.Pe/GII.17 could represent the first description of this recombinant strain.

Another recombinant strain detected in this study was GII.P16/GII.3, found in 2010 and 2011. In the same period, this recombinant was detected for the first time in Bangladesh, India and Italy [40,54]. The capsid genotype GII.3 was the second most frequent detected during a surveillance study carried out in southeastern Brazil in 2003–2004 [38]. In our study, the GII.3 capsid genotype was also detected as a recombinant strain associated with GII.P21 in 2008, but samples were grouped into different genetic clusters according to their polymerase genotypes. Both recombinant strains were also detected in Spain between 2009 and 2012, with GII.P21/GII.3 and GII.P16/GII.3 corresponding to 28.4% and 18.5% of all recombinant strains characterized, respectively [18].

In conclusion, we demonstrated the high diversity of recombinant strains causing AGE outbreaks in southern Brazil in 2004–2011, which represented the main percentage (85%) of non-GII.4 NoV. These data are in agreement with data obtained from a study conducted in Singapore, where only GII.6 and GII.7 capsid genotypes were non-recombinant viruses of all the non-GII.4 strains detected [49]. Our data show that the circulation of NoV recombinant strains is common in southern Brazil, with real potential ability to cause AGE outbreaks. The great diversity and the high frequency of recombinants circulating in our country demonstrates the importance of ongoing surveillance to understand the role of these recombinant strains in the dynamic of NoV infections. Inter or intra-genotype recombination allows increased fitness and viral evolution, enabling NoV to escape and spread in a susceptible population, with direct implications in NoV-infections incidence and for design of an effective vaccine. Therefore, the appropriate characterization of NoV strains is fundamental for performing an adequate epidemiological surveillance, highlighting the importance of a combined ORF1/ORF2 characterization to access the circulation and genetic diversity of NoV recombinant strains worldwide.
